# The Finances of Metropolitan Hospitals

**Published:** 1921-08-20

**Authors:** 


### The Finances of Metropolitan Hospitals.

### Within £2,000 of Guaranteed Overdraft.

As the bank overdraft of St. Paul's Hospital is within £2,000 of the £20,000 guaranteed by several members of the Committee, the outlay on the new premises in Endell Street will be restricted to about £700 a month for the next three months. It is hoped that at the expiration of that period the financial position will have improved sufficiently to enable construction to be expedited. The new premises are urgently needed, for the cramped quarters and the uncomfortable conditions under which the work is now done are unfair to both doctors and patients, and constitute a serious handicap to the remedial and preventive work of the institution. Up to the present £11,000 has been collected towards the £52,000 required. The Trustees of Smith's Charities sent £250.

### What St. George's is Doing.

The Committee of St. George's Hospital has expressed itself in favour of the system of "massed contributions," but has declined to adopt the "Sussex" scheme, as it considers that no attempt should be made to try such a scheme in London until it has been in operation for eighteen months. Moreover, it regards the estimate of the number of persons likely to avail themselves of the scheme as too optimistic. The hospital has been successful in its efforts to induce the Ministry of Pensions to date back to January 1, 1920, the increased rate of 11s. per day for in-patients, and the amount involved is £250. The financial position of the hospital is extremely disquieting; the deficiency on the year is expected to amount to £30,000, and the special efforts to avert this have so far been instrumental in obtaining only £1,300. If the full cost of treating Service men during the war had been paid by the Government, the deficiency would be £17,000 less. The innovation of patients' payments is held to have justified itself; during the first six months of the system £3,000 was received from in-patients and £1,500 from out-patients, as against a previous revenue of £400 from voluntary contributions from patients. The St. George's Hospital Work Society has been very active, and a Sale of Work organised by Lady English realised £100.

### Shadwell Hospital Extension Abandoned.

The East London Hospital for Children, Shadwell, has been compelled to abandon the extension project which, when launched in 1918, was estimated to cost £50,000. The expenditure of £6,000 on painting and repairs was absolutely essential, but even of this it has been possible to complete only a portion. Essential painting has been done and modern improvements installed in the kitchen quarters at a total outlay of £2,000. A cheering feature is the ungrudging character of local support; a recent flag day realised £900, no fewer than 600 flags being sold by three voluntary helpers before 10 a.m. The increased unemployment has had an adverse effect on box collections; boxes which at one time averaged £2 in a month now contain only £1 in six or seven months. The deficit revealed at the annual meeting was £11,000, and since then there has been a monthly deficit of about £1,000.

### S.O.S. for King's College Hospital.

It was the experience at King's College Hospital that the debt of £100,000 at the end of 1921 was in the first months of the current year increasing at the rate of £1,000 per week. With the greatest reluctance, and in order to prevent a worse calamity, the Committee of Management were compelled to close half the available beds for ordinary patients. The Committee of Management are now making a special effort to arouse greater interest in the hospital and its work, with a view to raising not only sufficient funds to liquidate the debt, but to enable them to reopen the seven closed wards. A gift from the makers of eight Roneo Duplicators is materially helping in the widespread appeal that is being issued.

